# Fortune telling: metabolic markers of plant performance

**DOI:** 10.1007/s11306-016-1099-1

**Published:** 2016-09-15

**Authors:** Olivier Fernandez, Maria Urrutia, Stéphane Bernillon, Catherine Giauffret, François Tardieu, Jacques Le Gouis, Nicolas Langlade, Alain Charcosset, Annick Moing, Yves Gibon

**Affiliations:** 1 UMR 1332 Biologie du Fruit et Pathologie, INRA, Centre INRA de Bordeaux, 71 av Edouard Bourlaux, 33140 Villenave d’Ornon, France; 2 Plateforme Métabolome Bordeaux, CGFB, MetaboHUB-PHENOME, 33140 Villenave d’Ornon, France; 3 UMR AgroImpact, INRA, Estrées-Mons, 80203 Péronne, France; 4 UMR LEPSE, INRA, Montpellier SupAgro, 34000 Montpellier, France; 5 UMR GDEC, INRA, UBP, 63039 Clermont-Ferrand, France; 6 UMR LIPM, INRA, CNRS, Université de Toulouse, 31326 Castanet-Tolosan, France; 7 UMR GQE, INRA, CNRS, Université Paris Sud, AgroParisTech, Ferme du Moulon, 91190 Gif-Sur-Yvette, France

**Keywords:** Breeding, Metabolic marker, Metabolomics, Plant performance, Prediction

## Abstract

**Background:**

In the last decade, metabolomics has emerged as a powerful diagnostic and predictive tool in many branches of science. Researchers in microbes, animal, food, medical and plant science have generated a large number of targeted or non-targeted metabolic profiles by using a vast array of analytical methods (GC–MS, LC–MS, ^1^H-NMR….). Comprehensive analysis of such profiles using adapted statistical methods and modeling has opened up the possibility of using single or combinations of metabolites as markers. Metabolic markers have been proposed as proxy, diagnostic or predictors of key traits in a range of model species and accurate predictions of disease outbreak frequency, developmental stages, food sensory evaluation and crop yield have been obtained.

**Aim of review:**

(i) To provide a definition of plant performance and metabolic markers, (ii) to highlight recent key applications involving metabolic markers as tools for monitoring or predicting plant performance, and (iii) to propose a workable and cost-efficient pipeline to generate and use metabolic markers with a special focus on plant breeding.

**Key message:**

Using examples in other models and domains, the review proposes that metabolic markers are tending to complement and possibly replace traditional molecular markers in plant science as efficient estimators of performance.

## Introduction

Forecasting the future is as old as the hills. How odd it might sound today but animals’ entrails, palm-reading and coffee grounds have been used in the past as a source of information by leaders and decision-makers. In modern society, we still need to anticipate. Proxy, diagnosis or estimation remain helpful for many human activities including scientific domains.

Metabolomics has recently taken a quantum leap forward. Using a combination of approaches such as proton nuclear magnetic resonance (^1^H-NMR), liquid or gas chromatography coupled with mass spectrometry (GC–MS, LC–MS) as well as robotized spectrometric and fluorimetric assays, it is now possible to measure thousands of analytes in thousands of samples whether of microbial, plant or animal origin (Gibon et al. 2012; Nicholson et al. 2007), even in non-model species. Metabolomics has a wide range of applications in an impressive list of organisms. For example, several ‘silent’ mutations in *Saccharomyces cerevisiae* bearing no overt phenotypes have been revealed by measuring metabolite concentrations (Raamsdonk et al. 2001). Metabolomics has also led to considerable progress in understanding the regulation of cellular metabolism in *Escherichia coli* (Nöh et al. 2007). In animal science, it has been used for studying the responses to adverse conditions in nematode and fruit fly (Coquin et al. 2008; Hughes et al. 2009; Malmendal et al. 2006) and for classifying the stages of embryogenesis in zebra fish by using fingerprints of highly correlated metabolites (Hayashi et al. 2009, 2011). Metabolomics is also widely used in edible products for predicting geographical origin, terroir and varietal effect, e.g. for wine (Cynkar et al. 2010; Tarr et al. 2013), green tea (Lee et al. 2015) and orange (Díaz et al. 2014), for assessing the legal requirements for oil, coffee, honey (Cubero-Leon et al. 2014) and for profiling the sensory qualities of wine and meat (Schmidtke et al. 2013; Straadt et al. 2014). Readers are referred to recent reviews on this subject (Cubero-Leon et al. 2014; Oms-Oliu et al. 2013; Putri et al. 2013; Sumner et al. 2015) for a more comprehensive view of these applications. The spread of metabolomics has been supported by increased computational power, which facilitates statistical analyses of large datasets and raises the possibility of applying correlative methods and finding metabolites associated with a given state or condition (Gibon et al. 2012; Wolfender et al. 2013). These so-called biomarkers can also be referred to as metabolic markers when constructed with metabolite concentrations.

Medical science has been precursor in the use of metabolic markers. Indian physicians around 1500 BC noted that the sugar-enriched urine of patients with diabetes attracted ants (Zajac et al. 2010). Nowadays, body fluid analyses offer numerous opportunities to profile metabolites and correlate them with a diagnosis and/or prediction of disease susceptibility. This is illustrated by the emergence of patient stratification and personalized medicine (Lindon and Nicholson 2014; Nicholson et al. 2012). Urine metabolic profiling led to the identification of metabolic markers of symptomatic gout (Liu et al. 2012) and preeclampsia (Austdal et al. 2015) and blood profiling has been used to estimate the risk of bacteremic sepsis in emergency rescue situations (Kauppi et al. 2016). Another promising application of metabolite analysis in medical science is the prediction of cancer risk (Lee et al. 2014; McDunn et al. 2013; Truong et al. 2013) or the evaluation of the putative effect of cancer treatments (Hou et al. 2014; Jiang et al. 2014; Wei et al. 2013).

Metabolic markers are also used in plant science. Early examples include diagnostic methods such as Jubil^®^ and N-tester^®^. They have both been used to proxy the nitrogen status in plants for the sustainable fertilization of wheat, barley and maize (Justes et al. 1997; Uddling et al. 2007) through measurements of nitrate in stem fluids or chlorophyll in leaves respectively. Because plant scientists and breeders are eager to improve crop performances in challenging conditions for human food security and to find varieties selected for more complex traits, metabolic markers are also becoming popular in plant science and breeding (Herrmann and Schauer 2013; Zabotina 2013). However, the use of metabolic markers is not straightforward. Metabolite levels belong to the phenotype, which means that they can be associated with the genotype, the environment, the developmental stage and the interactions between them, as any other trait. This might be why metabolic markers were first proposed as a tool for searching for metabolite quantitative trait loci (mQTLs) and finding the related genes (Fridman et al. 2000), which were subsequently used for selection. Nevertheless, metabolic markers can be used as direct predictors when associated with plant performance criteria. They can also contribute to understanding how plant physiology processes are co-ordinated in various growth conditions [e.g. as detailed for water deficit by (Tardieu et al. 2011)], although this may not be the primary objective, especially when using metabolic markers in breeding.

The aim of this paper is to define plant performance and metabolic markers and to explain why and when they can be used as a tool for monitoring or predicting such performance. Finally, we describe a cost-efficient pipeline using metabolic markers as putative predictors of performance, with notable applications in plant-breeding.

## What is plant performance?

The definition of crop performance is often limited to the yield of the harvested part of the plant bearing the added value. Yield is indubitably an important trait of performance and its pattern under various growth conditions may allow the simple comparison of genotypes. However practical, this definition of performance is partly inadequate. Performance traits can be qualitative such as behavior in a series of environmental scenarios (high temperature, water deficit or biotic stresses), crop subtypes (afila in pea, bearded wheat) or the association of traits that are desirable for a given crop. Additionally, crop performance can be related to an industrial procedure through which the crop has to be processed. We propose here a general definition of plant performance as being **an association of several traits that need to be monitored with regard to the plant life cycle or improved through a breeding process**. We propose the following non-exhaustive list of traits:

- Grain or tissue yield
- Stability and consistency of yield over various natural environments, meteorological conditions or stresses
- Plant morphology (number of leaves, stems, flowers per bunch, plant height…) or phenology (duration of a particular stage of development)
- Storage properties such as fruit shelf-life or grain stability
- Yield of a specific compound or metabolite (to increase its concentration or to eliminate it)
- Technological properties (e.g. malting properties for barley, protein quantity and quality for breadmaking in wheat, cooking properties for potato, etc.)
- Sensory quality such as the presence of aromas or aroma precursors
- Nutritional attributes such as absence or low content of anti-nutritional compounds, or presence of vitamins, glycemic index, saturated lipid content
- Tolerance to a specific adverse condition, biotic or abiotic stress (extreme temperatures, salinity…)
- Efficiency of water and nutrient use.

Several of these criteria are now included in large crop-breeding projects such as the French aMaizING (maize, www.amaizing.fr), BreedWheat (wheat, www.breedwheat.fr) and SUNRISE (sunflower, www.sunrise-project.fr) projects, which address a variety of agronomical objectives (e.g., tolerance to water stress, chilling, low nitrogen or sulphur availability) and include precise phenotyping. Biochemical or metabolic phenotyping are tentatively integrated into the breeding process, notably in order to establish more precise estimations of plant performance and access the underlying mechanisms.

## Definition of a metabolic marker

The term biomarker (or biological marker) originates from the field of medicine. It has been defined as ‘a characteristic that is objectively measured and evaluated as an indication of normal biologic processes, pathogenic processes, or pharmacologic responses to a therapeutic intervention’ (NIH Definitions Working Group, 2000). In plants, the concept of biomarker is often associated with plant performance and could be defined as **a characteristic that is objectively measured or evaluated as a predictor of plant performance**.

Biomarkers can be genotypic (e.g., nucleotide polymorphisms such as single-nucleotide polymorphisms or SNPs generally) or phenotypic (e.g., transcript levels, protein levels, enzyme activities, metabolite levels, images in different wavelengths). In addition to being predictive, biomarkers are preferably easy and cheap to score (Aronson 2005). This is probably why the use of molecular and biochemical markers, which proved to be excellent predictors and are relatively easy to measure in high-throughput conditions, became widespread in medicine (Menard et al. 2013; Robinette et al. 2013).

**Metabolic markers are a sub-category of biomarkers** that are involved in metabolism. Importantly, unlike DNA sequences, most metabolic traits vary during plant development, potentially with diurnal patterns, between tissue/organ and in response to environmental cues. Therefore, their use as biomarkers has to take into account developmental stage, position on the plant, time of day and growth scenario. Three types of metabolic markers can be distinguished:

- Traits of agricultural importance. An obvious strategy is to screen germplasm with direct measurements of such molecules or their precursors. Such traits can be desirable, like vitamin C or aromas (Ruiz-García et al. 2014; Pissard et al. 2013), or undesirable (e.g., toxins such as cyanogenic glucosides in cassava, anti-nutrients such as erucic acid in rapeseed).
- Diagnostic markers. In plants, single metabolic markers have been proposed to estimate the intensity of a given stress, for example proline, which accumulates in many species experiencing drought (Dib et al. 1994; Hayat et al. 2012). More recently, the idea that combinations of metabolic variables could be used to diagnose stress damage or resistance has emerged and the use of transcripts (Tamaoki et al. 2004), enzymes (Gibon et al. 2004) or metabolites (Korn et al. 2010, 2008; Roessner et al. 2000) has been proposed.
- Markers of genotype performance. In 2007, metabolic profiles were used for the first time to estimate biomass production in the model plant species *Arabidopsis thaliana*, with a coefficient of correlation of 0.58 (Table 1). This pioneering study paved the way for several others where associations between performance traits and metabolic markers were found, as summarized in Table 1. It also opened up new possibilities for plant breeding in which metabolic markers would be used to search for combinations of alleles that provide higher plant performance (Meyer et al. 2007). Ultimately, this would consist in searching for associations (e.g. with correlation, regression or classification methods), in a given set of genotypes, between metabolite data obtained for a given organ, developmental stage and environment combinations and plant performance, and then assuming that these associations remain valid for any genotypes grown subsequently in other environmental conditions.

**Table 1:** List of associations of metabolic markers and plant performance in recent literature

Plant	Trait	Numbers of markers	Statistical approach	Association	Statistical validation	Reference
Arabidopsis	Biomass	181	CCA/PLS	corr = 0.73/0.58	Random permutation/train-test sets	Meyer et al. 2007
	Dry weight	181	OLS/PLS	Q2Y = 0.11 (C24) and 0.11 (Columbia)/Q2Y = 0.12 (C24) and 0.23 (Columbia)	Random permutation/random permutation	Steinfath et al. 2010a
	Dry weight	9 (Columbia) 13 (C24)	PLS	Q2Y = 0.26/0.38	Random permutation	
Barley	Several malt quality traits	216	O2PLS	Q2Y = 0.17 to 0.79	–	Heuberger et al. 2014
Maize	Several performance traits	130	RR-BLUP	r(ĝ,g) = 0.61 to 0.79	Fivefold cross validation	Riedelsheimer et al. 2012a
	Dry matter yield	7	Pearson correlation	corr = −0.35 to 0.12	*p* value < 0.05	Riedelsheimer et al. 2012b
	Lignin content	7	Pearson correlation	corr = −0.20 to 0.15	p value < 0.01	
	Plant height	5	Pearson correlation	corr = −0.23 to 0.16	p value < 0.008	
	GCA for several performance traits	1/563	Pearson correlation/RR-BLUP	corr = −0.54 to 0.48/r(ĝ,g) = 0.47 to 0.78	Fivefold cross validation	Riedelsheimer et al. 2013
	Grain yield under drought stress	5	Pearson correlation	corr = −0.47 to −0.54	p value < 0.01	Obata et al. 2015
Pine	Plant height	11	Pearson correlation	corr = 0.13 to 0.35	p value < 0.05	Kang et al. 2015
	Stem dry mass	11	Pearson correlation	corr = 0.15 to 0.34	p value < 0.05	
Potato	Chip property	2	PLS and VIP selection	0.66 to 0.75	Random permutation	Steinfath et al. 2010b
	Susceptibility to blackspotedness	5	PLS and VIP selection	0.53 to 0.82	Random permutation	
Rice	Tolerance to mild salinity stress	2	t-test(foldchange)/PLS-DA	Delta log2(FCh) > 1/Q2Y = 0.49	p value < 0.05/random permutation	Nam et al. 2015
	Yield under drought stress	16	Pearson correlation	corr = −0.71 to 0.53	p value < 0.05	Degenkolbe et al.^2013^
	yield under drought stress	5	Pearson correlation	corr = −0.72 to 0.45	p value < 0.05	
Tomato	Firmess and shelf life	2	Correlation network	–	p value < 0.001	López et al. 2015
	TYLCV resistance	120	FCH/Correlation network	0.88 to 1.43/mean r2 = 0.62	p value < 0.05/–	Sade et al. 2015
Wheat	Fusarium graminearum resistance	60	FCH/Correlation network	–	–	Cuperlovic-Culf et al. 2016
Grape	Esca disease sensitivity	34	PCA	–	–	Lima et al. 2010

*CCA* canonical correlation analysis, *corr* correlation, *FCH* fold change, *GCA* general combining ability, *O2PLS* orthogonal partial least squares projections to latent structures, *OLS* ordinary least squares, *PCA* principal component analysis, *PLS* partial least squares to latent structures, *Q2Y* cumulative predictive explained variation, *r(ĝ,g)* correlation between predicted and unobserved true values, *RR-BLUP* ridge regression-best linear unbiased prediction, *VIP* variables importance in the projection

## Why use metabolic markers?

Measuring metabolites implies destructive sampling and sometimes costly and labor-intensive analytics. Furthermore, the use of molecular markers such as single nucleotide polymorphisms (SNPs), which are cheap, independent of the environment, amenable to high-throughput and are now commonplace in the research departments of breeding companies, is becoming the standard for breeders. So what would metabolic markers be good for?

### When metabolite levels are the trait of performance

Some metabolic traits are important *per se*. A famous example is zero-erucic-acid rapeseed oil, which is suitable for human nutrition. It was obtained with a strategy involving the non-destructive sampling of single cotyledons (to guarantee seedling survival to form the next generation) and quantification via gas liquid chromatography (Downey and Harvey 1963). Cyanogenic glucoside content in cassava, an important food source in tropical regions, could be measured by a low-cost spectroscopic method after acid hydrolysis (Bradbury et al. 1991) and then used in classical breeding programs aiming at reducing toxin levels (Nambisan 2011). Similarly, low phytic acid content in maize kernels is of interest for food and animal feed (Hazebroek et al. 2007). The screening of desirable metabolites is also possible, e.g. nutritional compounds such as vitamin C (Pissard et al. 2013) or aroma precursors such as rose oxide, which highly correlate with the “Muscat Aroma” in the grape cultivar (Ruiz-García et al. 2014). The role of metabolomics in improving the nutritional values of crops has already been underlined in rice (Fitzgerald et al. 2009) and these approaches could be a way to ensure that plant breeding programs place more emphasis on nutritional optimization (Anonymous 2016b).

### When metabolites provide condensed information

So far, most of the molecular marker–trait associations found in academic programs that have been transferred to commercial breeding programs involve traits with simple genetic determinism (Heffner et al. 2009; Xu and Crouch 2008). This is probably due to the fact that the number of molecular markers was initially low in most cases. Additionally, qualitative traits (disease resistance mostly) are overrepresented (Gupta et al. 2010). Furthermore, pyramiding beneficial alleles associated with traits resulting from complex interactions such as epistasis and genotype by environment interactions is still considered as very challenging (Furbank and Tester 2011).

In 2012, Riedelsheimer et al. (2012a) compared the predictive power of metabolic and molecular markers. Although the precision was slightly lower for metabolites with correlations ranging from 0.60 to 0.80 (Table 1) compared to 0.72 to 0.81, the authors underlined the fact that 130 metabolites were almost as good predictors as 38,000 SNPs. They concluded that metabolites provide condensed information and could be especially interesting when dealing with highly polygenic traits.

Two further studies in maize used a similar approach. The lipid profiling of maize leaves revealed high correlations with several agronomical traits [Riedelsheimer et al. (2013), including dry matter yield (0.47) and flowering time (0.78); Table 1]. A tempting follow-up would be to identify highly efficient hybrids in test-crosses via lipidomics. Caffeic- and p-coumaric acid also showed significant correlations with dry matter yield [−0.28 and 0.12 respectively; Table 1; Riedelsheimer et al. (2012b)], suggesting that a low-cost strategy targeting these metabolites could be developed to screen thousands of hybrids for selection purposes. In these examples, there is little difference in dealing with metabolic markers compared to molecular markers. Associations between metabolic markers and performance criteria would nevertheless have to be generated with adequate statistical methods that take into account potential interactions, e.g., between genotype and environment.

### When metabolites open the way to mechanistic insights

The fact that metabolic markers provide biological information that can narrow down the genotype-phenotype gap opens the door for mechanistic insights, starting with the detection of SNPs or candidate genes via mQTL mapping strategies. Riedelsheimer et al. (2012b) detected several mQTL for lignin precursors such as p-coumaric acid and caffeic acid, which they found to be good predictors of a range of plant performance criteria (e.g., plant height and dry matter yield; Table 1). The corresponding region harbors a key enzyme in monolignol synthesis (cinnamoyl-CoA reductase) and has been proposed as a good target for improving the quality of lignocellulosic biomass. In addition, candidate gene allelic variability (natural or induced) could be explored to evaluate changes in lignocellulosic quality. The use of metabolic markers to gain mechanistic knowledge can also be illustrated by the negative correlation of starch with biomass (Sulpice et al. 2009). This led the authors to conclude that starch is an integrator of plant growth, reflecting a fine balance between carbon supply and growth.

Such findings highlight the usefulness of metabolic markers for estimating agronomical traits and revealing biological mechanisms underlying phenotypes.

### When metabolites can be a diagnostic tool in crop processing

An original application of metabolic markers is the evaluation of crop performance in an industrial or commercial process. One of the first publications to mention such a possibility was focused on potato susceptibility to black spot bruising (induced by collisions during transport and storage) and undesirable ‘browning while frying’. Five amino acids (tyrosine, threonine, valine, serine and glutamine) and two sugars (glucose and fructose) were detected as the best metabolic markers (VIP in a PLS analysis; Table 1) for these traits, respectively (Steinfath et al. 2010b). To validate these markers, a model was entrained to compare measured and predicted traits in an independent location bearing significant correlation (ranging 0.53 to 0.82 and 0.66 to 0.75 respectively for susceptibility to blackspottedness and chip property; Table 1). Another example of metabolites linked to industrial properties is the association of a profile of 216 features (Table 1) to malting quality in barley (Heuberger et al. 2014).

Fresh fruit marketability is linked to shelf-life, which is affected by firmness. Both traits have been shown to be associated with malate content in tomato (López et al. 2015) through a neural network approach (self-organizing maps; Table 1). In the same study, another important commercial trait (fruit morphology) showed to be associated strongly with aspartate, glutamate and 2-oxoglutarate (López et al. 2015).

### When assessing diversity of crop core collections or other genetic resources

A recent application of plant metabolomics that has already been implemented in biotechnology and seed companies is the assessment of metabolic diversity within their crop core population or genetic lineage. This has been done for instance by Monsanto^®^ in soybean (Kusano et al. 2015; Harrigan et al. 2015) and maize (Venkatesh et al. 2016) as well as by Pioneer^®^ in the latter species (Baniasadi et al. 2014; Zeng et al. 2014; Asiago et al. 2012). Authors underline the potential of metabolomics to separate genetic and environmental effects on crop diversity (Venkatesh et al. 2016; Baniasadi et al. 2014) or for substantial equivalence studies of genetically modified (GM) genotypes (Harrigan et al. 2015; Baniasadi et al. 2014; Asiago et al. 2012). These results could be used to improve acceptance of GMOs and might also be used for regulatory purposes (Zeng et al. 2014). These companies have all the necessary tools in house to use metabolic data for breeding. Indeed several of their publications have already shown associations of key performance criteria with metabolites, for instance for yield in soybean (Kusano et al. 2015) or plant and ear height in maize (Venkatesh et al. 2016).

### When working on impact of abiotic and biotic stress

Metabolites can also be used as markers to estimate plant performance under stress conditions (Feussner and Polle 2015; Fraire-Velázquez and Balderas-Hernández 2013). Obata et al. (2015) found that myo-inositol accumulated in young leaves was constitutively and negatively associated with grain yield under at least some drought stress scenarios in maize (−0.54; Table 1) In rice, Quistián-Martínez et al. (2011) identified trehalose as a putative inducible marker in drought-tolerant rice genotypes, while Degenkolbe et al. (2013) reported eight metabolites that were positively accumulated in drought-tolerant varieties (including allantoin, galactaric and gluconic acid, glucose and salicylic acid glucopyranoside; Table 1). Interestingly, allantoin was also associated with salt-stress tolerance in rice (Table 1; Nam et al. 2015). Although ‘constitutive’ metabolic markers, e.g. those measured in plant material obtained under standard conditions and at young developmental stages (Riedelsheimer et al. 2012b; Riedelsheimer et al. 2012a), might be of great interest when stress resistance can be estimated, it is likely that ‘inducible’ metabolic markers will be needed to evaluate tolerance in stressed conditions and to train the prediction models of resistance. For this, the combined use of phenotyping platforms (Tisne et al. 2013) providing reproductive and relevant stress scenarios combined with pertinent metabolic analyses could be very valuable. However, such a strategy involving ecophysiologists, biochemists and geneticists still requires sustained exploratory efforts.

Regarding biotic stress, metabolomics has recently emerged as a tool for studying plant immunity, especially for deciphering the role of small molecules involved in plant–microbe interactions (Feussner and Polle 2015). Diagnostic-like strategies separating diseased from healthy plants with metabolic markers have been proposed using ^1^H-NMR in ornamental periwinkle and grapevine (Table 1; Choi et al. 2004; Lima et al. 2010). Finally, metabolic markers have been associated with tolerance to yellow leaf curl virus in tomato (Sade et al. 2015) and to fusarium in wheat (Cuperlovic-Culf et al. 2016). Of particular interest in the tomato study, the authors highlighted a more coordinated response of the primary metabolism in resistant cultivars (Sade et al. 2015).

## What pipeline to work with metabolic markers of plant performance?

The major challenge when using metabolic markers will be to establish combinations of growth scenarios, sampling strategies and metabolic marker measurements that provide estimations of plant performance that are consistent with the ‘real’ world. As mentioned above, it is indeed known that QTL associated with plant performance can have positive effects under given growth scenarios and negative effects under others (Tardieu 2011), and that there is a priori no reason why this would not be the case for such estimations. Vast numbers of metabolic fingerprints can be generated by profiling diverse organs or tissues at different stages and under various growth conditions. The fact that this diversity is challenging when looking for metabolic markers of performance implies that several steps listed below have to be taken into account.

### Growth scenarios: reproducible and crop-adapted to reveal diversity

Metabolite levels and fluxes are sensitive to growth conditions, especially to temperature which modifies enzymatic activities independently (Strand et al. 1999; Parent et al. 2010). They are also subject to large changes throughout plant and organ development and even throughout night and day cycles. Simulating the diversity of scenarios that any crop would face in the field is not a realistic option. Therefore, careful implementation of reproducible growth scenarios seems necessary to find the best metabolic markers, especially if the studied performance criterion is tolerance towards adverse conditions.

These scenarios should be designed in order to reveal genotype diversity for a given plant performance criterion. They can be seen as a proxy of the growth conditions of the crop with the additional constraint of reproducibility in order to generate robust markers. Academic (Cabrera-Bosquet et al. 2016; Kumar et al. 2015) and private robotized phenotyping facilities offer solutions for programming such scenarios and for phenotyping crops while limiting costs compared to field trials (Humplík et al. 2015). These facilities, which so far tend to focus on growth and architecture, could be used to perform metabolic studies, eventually identify metabolic markers and ultimately deepen our knowledge about how metabolism and plant performance are integrated. It is likely that this will require large experimental (e.g., what should be harvested, at what developmental stage, at what time of the day, what should be measured) and technological (e.g., cost-efficient sample collection) efforts.

In association with this type of facilities, data and metadata management solutions (Hannemann et al. 2009) would be of great help. Indeed, the extensive follow-up of experimental conditions (detailed scoring of all environmental and developmental factors that may impact metabolism…) from growth scenarios to sample handling and metabolomics data, would greatly facilitate the integration of such factors with plant performance and help in generating accurate metabolic markers.

### Sampling procedure: easy to harvest and process

Wen et al. (2015) studied the predictive power of metabolomic data obtained from different organs/stages for agronomical traits in a maize population (leaves at seedling and reproductive stages and kernels at 15 days after pollination). Only 33 of the 79 identified metabolites were commonly detected between these organs/stages and the evaluated agronomical traits were predicted by different combinations of metabolites depending on the sampling matrix. Metabolic marker selection might therefore be conditioned by both the organ/tissue and the developmental stage at sampling time, and also largely depend on the trait studied. Pragmatically, metabolic markers would be sought at young developmental stages first in order to reduce screening costs, and in leaves, which are easy to collect, handle and analyze. Furthermore, it seems logical that the later the samples are taken during development, the greater the chances of finding good correlations between metabolite levels and traits of interest. Thus, taking samples as early as possible in plant development would result in robust prediction and metabolic markers. Finally, the best option for each case needs to be carefully evaluated and pondered considering the expected results and required investment.

### Number of metabolic markers vs sample size: finding the right balance for cost efficiency

Although targeted metabolite profiling by electrospray ionization tandem mass spectrometry allows hundreds of metabolites to be measured in thousands of samples for human Genome-Wide Association Studies (Gieger et al. 2008), in depth metabolomics remains too costly for the analysis of very large numbers of plant genotypes (ranging from 30 to 300 € per sample; Gibon et al. 2012). In other words, when looking for associations with plant performance, ‘metabotyping’ every genotype appears to be impossible at a reasonable cost so subpanels have to be designed. Subpanel selection is rarely described in detail. One possibility is to maximize diversity based on phenotypic or molecular data (Rincent et al. 2014). The constitution of bulks of extreme genotypes has been widely used for genomics (Zou et al. 2016) and has been successfully tested for metabolic data (Zhang et al. 2010). Numerous sampling survey methods exist (Singh and Singh Mangat 1996) but their adaptability to plant metabolomics data is uncertain and has received little attention to date. We foresee two possible non-mutually exclusive options for in depth metabolomics analysis:

- Untargeted metabolic phenotyping in diversity subpanels

Subpanels of highly diverse genotypes and/or given growth scenarios could be investigated first by using non-targeted analytical approaches and identifying the best markers, thus keeping costs acceptable by reducing the sample number. The number of potential metabolic markers generated via untargeted analysis could then be reduced by selecting those that provide good discrimination between genotypes, environments and their interactions, on the one hand, and which are easily amenable to high-throughput on the other. Targeted methods would then be developed to characterize the full panel and/or the full set of growth conditions. If the metabolic marker has been generated through LC–MS technology, the development of a targeted method requires accurate annotation of the compound. Readers are referred to (Wolfender et al. 2015) as a guideline for annotation in complex extracts.

- Targeted measurements

Such measurements should enable high numbers of samples to be processed at low costs, thus enabling screens of large populations and/or complex experimental setups (diverse growth scenarios, developmental stages, etc.). For example, LC–MS targeted profiles could be generated automatically at moderate cost (50–100 € per sample; Heuberger et al. 2014). Sample preparation and equipment investment still account for a large part of LC–MS analysis costs and they can both be improved by automation and increase in throughput (de Raad et al. 2016; Novakova 2013). The cost of data handling, curation and analysis also has to be taken into account (Anonymous 2016a).

High-throughput spectrophotometric analysis of major sugars and organic acids, which are respectively powerful predictors of potato quality (Steinfath et al. 2010b) and tomato (López et al. 2015), could be easily implemented in facilities using robotized microplate measurements (Ménard et al. 2014) and for less than 20 € per sample. However, for many volatile compounds and secondary metabolites, there will still be certain limitations to reducing costs by methodologic adaptations (Kallenbach et al. 2014), although future developments may offer new possibilities.

### Data analysis for modeling plant performance: custom-made solutions

Detection of markers is linked to the idea of associating explanatory variables (X, markers) and response variables (Y, targeted phenotype). Therefore, an appropriate statistical method estimating such an association between metabolites or metabolite signatures and phenotypic variables and its significance is necessary.

In the simplest scenario where one metabolite is highly correlated to the targeted phenotypic trait, a pair-wise Pearson’s correlation might be sufficient to detect an appropriate marker. However, a more likely situation is that more than one metabolite is needed to build a predictive model. In such cases, some commonly applied statistical methods are used to maximize the correlation between X and Y. Among them, canonical correlation analysis (CCA) estimates the maximum correlation between linear combinations of X and Y matrices, while stepwise regression and best subset regression aim at maximizing the correlation by selecting a minimum number of variables in X that predict Y (Song et al. 2016). Other very widespread methods are used to maximize covariance. If genotypes can be easily grouped in a few clusters based on their agronomical performance(s), these groups can be used to search for biomarkers using discriminant analysis. Partial Least Square Discriminant Analysis (PLS-DA) maximizes covariance between X and Y, thereby reducing the explanatory variables to a set of PLS components whose optimal number is selected by cross-validation. PLS methods have the advantage of handling highly collinear and noisy datasets (Wold et al. 2001), as is the case for most metabolomics data sets. A variant of PLS, Orthogonal Partial Least Squares (OPLS), reduces the noise effect by splitting variation in X matrix between correlated (predictive) and uncorrelated (orthogonal) to Y. This orthogonal signal correction aims at maximizing the explained covariance between X and Y on the first OPLS component while the subsequent components explain the uncorrelated variance to Y (Trygg and Wold 2002). (O)PLS statistical validation is performed by random permutation of labels and by dividing the samples into two random groups, one of them aiming to fit a model and the other to estimate its predictive power or quality. In addition, (O)PLS allows variable selection among X variables through several statistics, variable importance in projection (VIP) being the most commonly known but not the only one (Galindo-Prieto et al. 2014; Mehmood et al. 2012). Although these are very popular methods in metabolomics, there are other appropriate alternatives like principal component-discriminant function analysis, support vector machines and random forest (Gromski et al. 2015). All the above multivariate methods are prone to overfitting, so validation with a different dataset from the one used to fit the model is mandatory.

A possibility is to begin a metabolic marker search process using the following workflow. Normalization has to be done first according to data scale and heteroscedasticity (van den Berg et al. 2006). Log 2 normalization is often preferred for univariate analysis, whereas Z-score or Pareto normalization is done before multivariate analysis. The data matrix is first analyzed with a univariate method (e.g. one or two-factor ANOVA, possibly genotype and treatment) to obtain the most significant metabolites affected by each factor and to check whether genotype x treatment interactions are present. Some highly correlated variables may also be removed at this stage to improve further modeling. Multivariate unsupervised analyses (PCA) are generally performed to give a global snapshot of the data and check for outlier samples. Finally, supervised methods such as PLS-DA and OPLS-DA are carried out. They provide VIP values that can be used to select potential candidates for metabolic markers. In parallel, machine learning methods (random forest, neural network…) might be applied but their use is still limited in plant metabolomics. Note that this analytic procedure is given as a basic guideline and should be adapted for each target and type of data matrix, then complemented with other statistical methods.

### The example of plant breeding

**Fig. 1:**
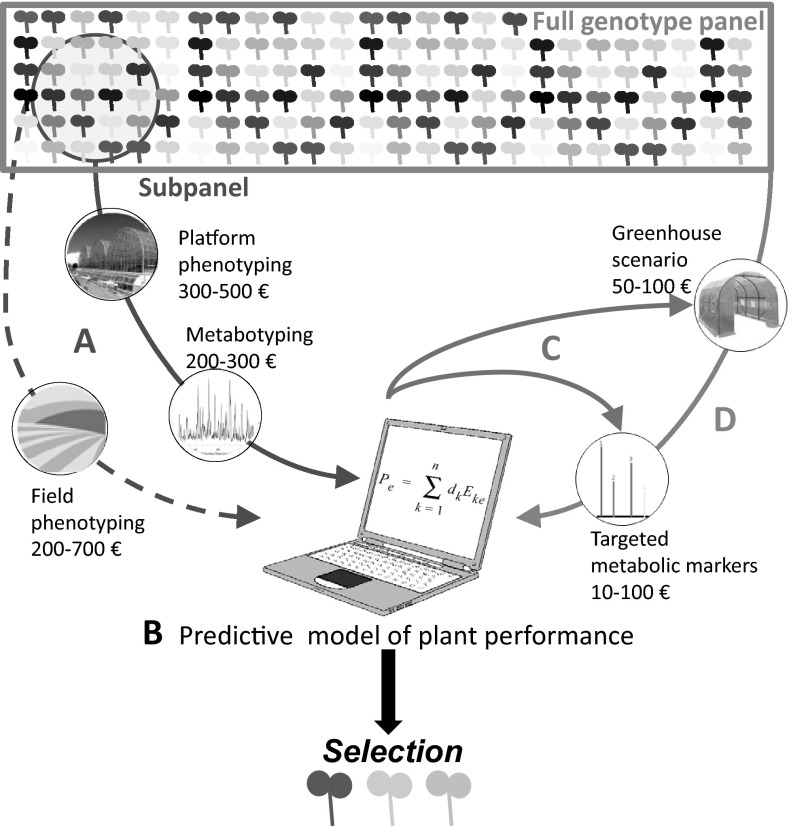
Strategy combining phenotyping, metabotyping and modeling for selection in order to find a few performing genotypes from a full panel of genotypes for a given criterion. Metabolic marker may optimize cost and speed of the process by (*A*) “metabotyping” and precision phenotyping of a diversity subpanel in a series of representative environmental conditions, (*B*) using collected data to model genotype performance. The model would generate a workable combination of (*C*) adapted growth scenario, sampling procedure and a small cost-efficient set of metabolic markers which would be used for (*D*) validation on the full panel of genotypes or for a further selection program. For the purpose of estimating costs, we consider 1 sample per genotype as a pool of 5 plants

To illustrate and summarize the search for and use of metabolic markers, an example of a pipeline for plant breeding is given in Fig. 1: (1) ‘Metabotyping’ of smaller representative subpanels of genotypes [see for instance Rincent et al. (2012) for discussion on panel sampling in a predictive context] is performed in parallel with acquisition of other phenotypic variables of interest in the field or on phenotypic platforms (Fig. 1A). (2) These data are used to train models estimating traits of interest (Fig. 1B) and aiming at optimizing growth conditions and sampling, and if possible, at reducing the number of metabolic markers (Fig. 1C). (3) With such optimization, a small set of metabolic markers (10–20 markers) can be measured at a cost of 10–100 € per sample in a breeding pipeline (as shown in Fig. 1; e.g. a pool of 5 individuals from the same genotype), making it possible to use them for full diversity panels (Fig. 1D). The estimated cost for use of a molecular marker is between 10 and 30 € per sample and they will continue to be improved thanks to sequencing technologies (Next-generation sequencing, Genotyping by Sequencing). Nevertheless, if the proposed pipeline is carefully followed, metabolic markers would be able to compete with molecular markers based on relevance rather than just on cost in certain situations.

## Conclusion

Metabolites have a great potential as markers of plant performance because they contain more information in certain scenarios and give a more realistic picture of ‘real’ plant performance than molecular markers. Indeed, leading biotech companies have already or are in the process of integrating these tools in their crop selection projects (Venkatesh et al. 2016; Baniasadi et al. 2014).

**Fig. 2:**
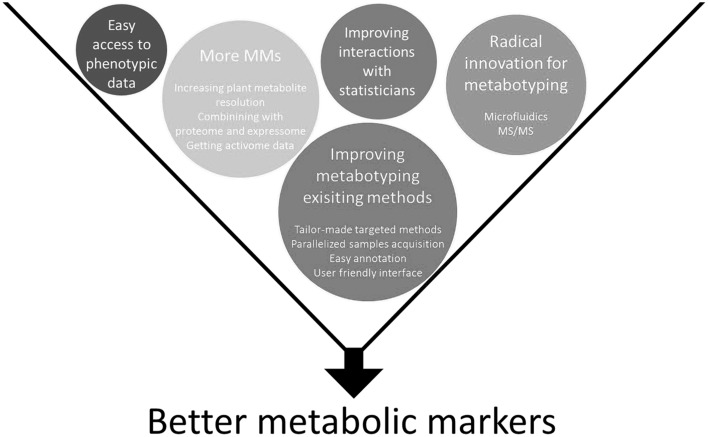
Key milestones for improving and developing the use of metabolic markers

However, if metabolic markers are to express their full potential, several technological breakthroughs will be needed (Fig. 2). Available analytical methods have to be democratized and made more user-friendly, especially the possibility of parallelizing sample flow and data acquisition (Deng et al. 2002). Furthermore, solvent quantities need to be reduced by using micro-fluidic devices (Gao et al. 2013) and tailor-made targeted methods able to measure 10–20 metabolic markers simultaneously need to be developed. Dedicated new methods with metabolite sensors using microfluidics could be used for plant samples, as is already the case in human health (Tharakan et al. 2015). In addition to the development of methods for the parallel measurement of individual small molecules such as ELAKCA (a sandwich-type enzyme-linked assay), breeding would benefit from a tunable platform in which such assays could be easily adapted to each specific marker (Chovelon et al. 2016). Methods targeting other types of metabolic markers such as transcripts or proteins could also be implemented. Thus, enzymatic activities could well prove to be efficient markers as well since they correlate poorly with metabolites (Sulpice et al. 2010) and would therefore add a new layer of information for modeling plant performance. Closer collaboration between statisticians and bioinformaticians is required and plant scientists need to become more familiar with advanced statistical methods.

Finally, phenotypic data on existing genotypes should be made more accessible because they offer a great potential for correlating or associating putative markers with known genotype performance. This is clearly the goal of the DivSeek consortium (Anonymous 2015) but other initiatives, be they public or private, should be fostered.
